# "First pain" in humans: convergent and specific forebrain responses

**DOI:** 10.1186/1744-8069-6-81

**Published:** 2010-11-17

**Authors:** Dagfinn A Matre, Luis Hernandez-Garcia, Tuan D Tran, Kenneth L Casey

**Affiliations:** 1 Dept. of Work-related Musculoskeletal Disorders, National Institute of Occupational Health, Oslo, Norway; 2 Dept. of Neurology, VA Medical Center, Ann Arbor, MI, USA; 3 Dept. of Neurology, University of Michigan, Ann Arbor, MI, USA; 4 Functional MRI Laboratory, University of Michigan, Ann Arbor, MI, USA; 5 Dept. of Pediatrics, University of Medicine and Pharmacy of Ho Chi Minh City, Ho Chi Minh City, Vietnam

## Abstract

**Background:**

Brief heat stimuli that excite nociceptors innervated by finely myelinated (Aδ) fibers evoke an initial, sharp, well-localized pain ("first pain") that is distinguishable from the delayed, less intense, more prolonged dull pain attributed to nociceptors innervated by unmyelinated (C) fibers ("second pain"). In the present study, we address the question of whether a brief, noxious heat stimulus that excites cutaneous Aδ fibers activates a distinct set of forebrain structures preferentially in addition to those with similar responses to converging input from C fibers. Heat stimuli at two temperatures were applied to the dorsum of the left hand of healthy volunteers in a functional brain imaging (fMRI) paradigm and responses analyzed in a set of volumes of interest (VOI).

**Results:**

Brief 41°C stimuli were painless and evoked only C fiber responses, but 51°C stimuli were at pain threshold and preferentially evoked Aδ fiber responses. Most VOI responded to both intensities of stimulation. However, within volumes of interest, a contrast analysis and comparison of BOLD response latencies showed that the bilateral anterior insulae, the contralateral hippocampus, and the ipsilateral posterior insula were preferentially activated by painful heat stimulation that excited Aδ fibers.

**Conclusions:**

These findings show that two sets of forebrain structures mediate the initial sharp pain evoked by brief cutaneous heat stimulation: those responding preferentially to the brief stimulation of Aδ heat nociceptors and those with similar responses to converging inputs from the painless stimulation of C fibers. Our results suggest a unique and specific physiological basis, at the forebrain level, for the "first pain" sensation that has long been attributed to Aδ fiber stimulation and support the concept that both specific and convergent mechanisms act concurrently to mediate pain.

## Background

There is substantial evidence that pain is mediated by two classes of nociceptive afferent fibers, finely myelinated Aδ fibers and unmyelinated C fibers [1,2]. Following a brief (< 1 sec) noxious cutaneous heat stimulus, two distinct sensations arise: an initial, sharp pain thought to be mediated by Aδ fibers ("first pain") and a delayed, less intense, more prolonged, heat sensation ("second pain") that is attributed to the excitation of C fibers [2,3]. These two pain experiences have unique psychophysiological and pharmacological characteristics [3-5], supporting a long-standing hypothesis that each fiber type activates central pathways that are anatomically unique although partially overlapping [2]. Functional imaging studies show that a number of brain regions are active during pain including the primary and secondary somatosensory cortex, insula, cingulate cortex and thalamus [6-8], but the relative contribution of each fiber type to the elicited BOLD responses are unknown. Electrophysiological studies, using magnetoencephalography, evoked potentials (EP), and selective laser stimulation of Aδ and C fibers show very similar locations of the early cerebral activations from these sources [7,9-11]. Qiu and colleagues used laser stimulation and functional magnetic resonance imaging (fMRI) to show that the cerebral activations following preferential Aδ fiber stimulation overlap those evoked by preferential C fiber stimulation although the C activations were significantly greater in the bilateral anterior insulae and the ipsilateral medial frontal gyrus [12]. The results reported by Qiu and colleagues [12] suggest an extensive anatomical overlap of structures activated by painful Aδ and C fiber stimulation; however, an anatomically unique aspect of Aδ-mediated pain remains in question. A related functional imaging study by Veldhuijzen and colleagues also revealed an extensive overlap of structures activated during equally intense sharp or burning pain following diode laser stimulation at parameters designed to favor Aδ or C fiber excitation, respectively [13]; however, some temporal and parietal lobe structures were more active during sharp pain while the dorsolateral prefrontal cortex responded more during burning pain. There is also uncertainty about whether the unique cerebral responses to the brief excitation of C fibers are related specifically to pain. This question arises because brief cutaneous heat stimulation above C, but below Aδ fiber threshold, is not reliably painful [12,14,15] and because cerebral structures activated by C fibers responding to warm or tactile stimuli [16] may also respond during Aδ-mediated pain.

In the present study, the aim was to identify the cerebral mechanisms that could be uniquely involved in mediating the brief, sharp "first pain" that is mediated by Aδ fibers. This was done by comparing responses to a noxious heat stimulus that excites cutaneous Aδ fibers with responses to converging input from C fibers. We used brain evoked potentials to identify the brief heat stimuli that preferentially excite Aδ and C fibers and we used functional magnetic resonance imaging (fMRI) to compare quantitatively the brain responses to each of these stimuli. We used contrast imaging and temporal analysis to detect the preferential activation of brain structures by Aδ fibers.

We found that two sets of forebrain structures mediate the initial sharp pain evoked by brief cutaneous heat stimulation. One set responding preferentially to the brief stimulation of Aδ heat nociceptors and those with similar responses to converging inputs from the stimulation of C fibers.

## Methods

### Subjects

Seven right handed volunteers (age 18-36 years old; three females) participated in the experiment. All subjects were free of medication and had not consumed caffeine or other psychoactive drugs on the day of testing. All subjects gave informed consent before testing. The experimental protocol was conducted in accordance with the Helsinki declaration and was approved by the Internal Review Boards of the Veterans Affairs Medical Center (Ann Arbor) and the University of Michigan, Ann Arbor, MI, USA.

### Somatosensory stimuli

We used a *contact-heat evoked potential stimulator *(CHEP stimulator; Medoc, Ramat Yishai, Israel). The thermode was strapped to the hand and not moved between stimuli. The thermode contacted a cutaneous area of 573 mm^2 ^and is comprised of a heating thermofoil (Minco Products, Inc., Minneapolis, Minnesota) covered with a 25 micron layer of thermoconductive plastic (Kapton^®^). Two thermocouples embedded 10 microns within this conductive coating provide an estimate of the skin temperature at the thermode surface. The thermofoil-skin interface temperature is measured and software-controlled 150 times per second. Heat stimuli were delivered at intensities of 41°C and 51°C. From a contact baseline temperature (35°C), the average time from onset to peak temperature is on average 190 ± 24 ms for 41°C and 250 ± 8 ms for 51°C. The full-width-half-maximum (FWHM) of the heat pulse is 350 ms. As reported previously, the stimulus parameters selected here reliably evoke quantifiable contact heat evoked potentials (CHEPs) mediated through C fibers accompanied by a warm sensation (41°C) or mediated through Aδ fibers accompanied by a sharp, pricking pain (51°C) [14,17]. With stimuli stronger than 41°C, an earlier potential associated with Aδ fiber excitation may begin to appear and the sensation is more like to be slightly painful (see Results). With stimuli less than 51°C, the positive potential associated with Aδ fiber stimulation and the sensation of pricking pain are less reliably present. Stimulation above this intensity is often associated with a delayed burning sensation suggesting the excitation of C fibers. Therefore, the stimulus parameters we used here for brief heat pulses were selected to emphasize the electrophysiological and psychophysical characteristics of "first pain" and maximize the combined electrophysiological and psychophysical differences between Aδ and C fiber excitation.

### Experimental protocols

**Figure 1:**
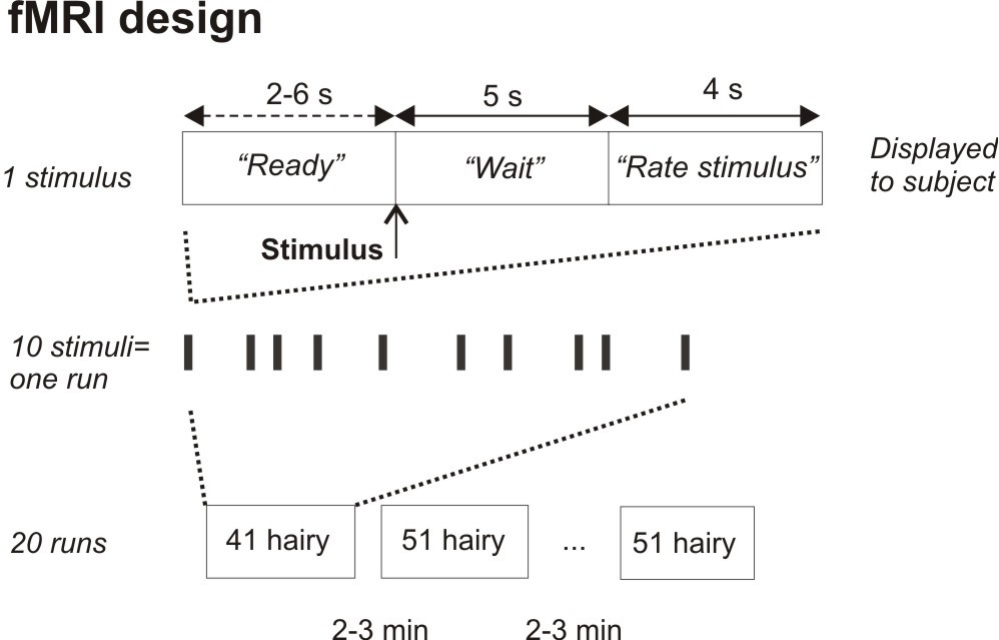
**Experimental protocol of fMRI experiment**. The top row show the messages displayed to the subject in the scanner for each somatosensory stimulus. The stimulus was delivered after a 2-6 s delay ("Ready" message), followed by a 5 s "Wait" message before a 4 s "Rate stimulus" message was displayed, at which time the subject rated the stimulus on a five-button claw. The middle row illustrates how one stimulus (bars) is repeated 10 times, creating one run. The bottom row illustrates how each run is repeated with different stimulus modalities, making a total of 20 runs. Only stimulus intensities delivered to hairy skin (41°C and 51°C) is reported here. Intermingled were two heat stimulus intensities delivered to glabrous skin and one epidermal electrical stimulus. Results from these stimuli are reported elsewhere.

Evoked potentials (EPs) and functional magnetic resonance images (fMRI) were acquired in two sessions one to four weeks apart. All subjects participated in both sessions and received up to 200 stimuli in each session; 20 runs of 10 stimuli divided into 4 runs of five different modalities (epidermal electrical, 41°C to hairy and glabrous skin, and 51°C to hairy and glabrous skin) (see Figure 1). Most subjects received fewer than 200 stimuli during the EP session, because two runs of each stimulus modality were usually enough to provide an easily identifiable brain potential. Responses to electrical stimuli and to heat stimuli delivered to glabrous skin will be reported elsewhere; hence the present article reports responses only to 41°C and 51°C heat stimuli delivered to hairy skin on the dorsum of the left hand. The inter-stimulus-interval (ISI) varied randomly between 11 and 15 s. The stimulus electrode/thermode was not moved between stimuli.

### Intensity ratings

The perceived intensity was rated after each stimulus, according to a 5-point numerical rating scale (NRS): 1 - 'not perceived', 2 - 'perceived but not painful', 3 - 'barely painful', 4 - 'moderately painful' and 5 - 'very painful'. Before data acquisition, the subject was instructed to pay close attention to the stimulus site and to rate the intensity of each stimulus approximately two seconds after it was perceived (EP) or when a visual cue appeared (fMRI). The ratings were performed verbally (EP) or via a five-button claw attached the right hand (fMRI). The five-button claw was a custom designed key pad strapped to the hand having one button for each finger (thumb = 1, little finger = 5). Pain intensity ratings and EP latencies were not normally distributed and therefore compared with the non-parametric Wilcoxon test. The Mann-Whitney U test was used for post-hoc comparisons; p < 0.05 was considered statistically significant. These statistical measures were carried out using SPSS (SPSS 15; SPSS Inc., Chicago, Illinois, USA).

### EP acquisition and analysis

Before recordings, the subjects were presented with the different stimuli and practiced subjective ratings. Recordings were made from the vertex (Cz), referenced to bilateral earlobes (A1+A2), using a standard EEG cap and Neuroscan software (Scan 4.2, Compumedics, El Paso, Texas). The electrooculogram (EOG) was recorded from supra- and infraorbital electrodes for offline artifact rejection. The impedance was maintained below 5 kΩ. The signals were amplified 100 000 times, sampled at 500 Hz, bandpass filtered at 0.1 - 30 Hz and notch filtered at 60 Hz. Subjects were seated in a padded reclining chair, room temperature was 22-23°C, and skin temperature was always above 30°C. The subjects were instructed to keep their eyes open, to focus on a fixed point at the wall and to avoid blinking. During offline analysis, the continuous EEG signal was split into epochs relative to stimulus onset using EEGLAB 6 [18]. Each epoch was visualized and discarded if contaminated by ocular artifacts. The remaining sweeps were averaged for each stimulus modality and subject. The peak latency of the major negative and positive component was identified by visual inspection of the averaged response. Peak latencies were compared with Wilcoxon paired non-parametric test (SPSS 15.0).

### fMRI image acquisition

Imaging was performed in a 3 Tesla GE Signa scanner using a standard 16 rung bird-cage head coil. The head was packed with foam pads and strapped with a headband to avoid head movement. In each run 96 whole T2*-weighed spiral-out brain scans were acquired (24 axial slices, 5 mm thick with no gap). The field of view was 24 cm with a 64 × 64 matrix. Repetition time (TR) was 1500 ms, echo time (TE) 24 ms and flip angle 60°. T1-weighed structural scans for anatomical localization (60 slices, 0.95 mm thick) were also obtained (field of view 24 cm, 256 × 256 matrix, TR 200 ms, TE 3.4 ms, flip angle 90°). The stimulation paradigm was controlled by computer software (E-prime, Psychology Software Tools, Pittsburg, Pennsylvania). The software also stored subjective ratings via the 5-button claw strapped to the subject's right hand. The computer running E-prime was triggered by the onset of the scanner radio frequency (RF) pulse. The computer then delivered trigger signals to the stimulator. Exact stimulus onset times were stored for later use during the statistical modeling.

Messages were presented to the subject via head mounted MRI compatible LCD display (Resonance Technologies, Los Angeles, CA). Before each stimulus, a 'READY' screen was displayed for 2 to 6 s. At the offset of 'READY' the stimulus was delivered. The following 'WAIT' screen lasted 5 s before 'RATE STIMULUS' was displayed for 4 s, at which time the subject rated the stimulus by pushing one of the five buttons (Figure 1). After each run there was a 2-3 min break. The modalities were presented in a pseudorandom sequence between runs. To maintain the subject's alertness, they were told that over one run the stimulus intensity could vary slightly, or could be the same, and that a purpose of the study was to determine their ability to detect this.

### Definition of volumes of interest (VOI)

Based on the previous experience in this laboratory and the results of published pain activation studies [6,7], volumes of interest (VOI) were defined anatomically as boxes, based on the brain anatomy atlas by Talairach and Tornoux [19]. VOI included the primary and secondary somatosensory cortex (S1/S2), anterior and posterior insula (AIns/PIns), pregenual -, anterior mid-, posterior mid-, and dorsal posterior cingulate cortex (pACC, aMCC, pMCC, dPCC), hippocampus (Hippo), thalamus (Thal), hypothalamus (Hypo), and posterior orbitofrontal cortex (POFC) bilaterally (the latter delineated within a 13-mm radius sphere). For the cingulate cortex, we adopted the anatomical terminology used by Vogt [20]. Several structures were divided into two or more VOI (e.g. Hippocampus). Table 1 presents the min-max Talairach coordinates in each direction for the 36 VOI. Negative x-coordinates are ipsilateral to the stimulus (left side of brain). A binary image (mask) was generated for each VOI.

**Table 1:** Volumes of interest (VOI)

			**x (+/-)**		**y**		**z**		**#**
			**med**	**lat**	**post**	**ant**	**inf**	**sup**	**voxels**
	
1	S1 - lateral	LS1	32	52	-41	-22	40	60	950
2	S1 - medial	MS1	10	31	-41	-22	40	60	998
3	S2	S2	45	60	-20	-8	10	20	225
4	Pregenual ant. cingulate cortex	pACC	1	10	33	48	-5	20	422
5	Anterior midcingulate cortex	aMCC	1	10	0	33	20	40	743
6	Posterior midcingulate cortex	pMCC	1	10	-25	0	30	40	281
7	Dorsal post. cingulate cortex	dPCC	1	10	-42	-25	10	40	574
8	Anterior insula	A Ins	25	40	0	18	-6	18	810
9	Posterior insula	P Ins	30	45	-22	-1	-6	18	945
10	Posterior orbitofrontal cortex*	POFC	17	43	15	41	-17	9	1150
11	Thalamus - medial	MThal	0	10	-32	-2	0	16	600
12	Thalamus - lateral	LThal	10	22	-32	-2	0	16	720
13	Hypothalamus - superior	HySup	0	15	-12	0	-8	-4	90
14	Hypothalamus - inferior	HyInf	0	10	-7	0	-12	-9	26
15	Hippocampus - superior	HippSup	10	30	-50	-30	-4	4	400
16	Hippocampus - low superior	HippLowSup	10	35	-40	-26	-12	-5	306
17	Hippocampus - high inferior	HippHiInf	17	38	-25	-8	-20	-13	312
18	Hippocampus - inferior	HippInf	17	35	-20	-5	-24	-21	101

The six Talairach coordinates (in mm) defining each volume of interest (VOI) and the approximate number of voxels per VOI. med: medial; lat: lateral; post: posterior; ant: anterior; inf: inferior; sup: superior. x-coordinates are bilateral, '+' is right and contralateral to stimulus; '-' is left and ipsilateral to stimulus, making 36 VOI. * Coordinates are min/max of a 13-mm radius sphere centered at at +/- 30 28 -4. Coordinates are based on the stereotactic atlas by Talairach & Tournoux [19].

### Group level analysis of main effects and contrasts between intensities

The volumes were motion corrected (MCFLIRT) and spatially smoothed (5-mm FWHM Gaussian kernel) and high-pass filtered (50 s). General linear model analysis was carried out using FEAT (FMRI Expert Analysis Tool) Version 5.4, part of FSL (FMRIB's Software Library) [21]. The trials in each run were modeled as delta functions convolved with the gamma function and its first derivative, as implemented in FSL. The first three volumes in each run were deleted to achieve steady-state magnetization. The contrast images were then spatially normalized to MNI-space (2.0, 2.0, 2.0 mm) and analyzed in a higher-level mixed-effects analysis. Contrasts were defined for main effects of stimulus intensity (41 and 51) and for differences between stimulus intensities (i.e. 41 < 51 and 41 > 51). Higher-level analysis was carried out using FLAME (FMRIB's Local Analysis of Mixed Effects) [22,23]. Finally, for each contrast, a one-sample T-test was performed on the results of the mixed effects model using SPM2 [24]. Significant activations were calculated at group level within each of the a priori defined VOI. The VOI masks were used as search volume when activation maps were assigned a statistical threshold of p < 0.01, corrected for multiple comparisons by using the theory of random fields [25] and SPM2's small-volume correction (S.V.C.) function. Within each VOI, the number of suprathreshold (p < 0.01, S.V.C. corrected) voxels is reported, in addition to the Z-score and Talairach coordinates of the most significant voxel.

### Time series analysis

For each VOI and stimulus intensity, single trial averages were calculated from the individual BOLD responses. First, an average BOLD response of the significant voxels (p < 0.01, uncorrected) was calculated across the four runs per stimulus intensity for each subject. Second, an average BOLD response was calculated across subjects, resulting in 72 BOLD responses (36 VOI × 2 stimulus intensities). The BOLD responses were extracted from the functional images in subject space by inverse transformation of the 36 VOI mask images. Voxelwise normalization over the time course was achieved by dividing the signal intensity at each time point by the voxel's mean intensity. All calculations were done using custom made Matlab software (Matlab 7.0, The Mathworks Inc., Natick, MA, USA) [26]. We identified the peak latencies of each BOLD response in order to categorize VOI that showed an Aδ-predominant response from VOI that responded to Aδ- and C-fiber input. A later peak latency was taken as a contribution from C-fibers. Thus, VOI were defined as differentiating between Aδ and C fiber inputs if the peak BOLD latency at 51°C stimulation was shorter than the peak BOLD latency at 41°C stimulation.

A major difference between the time series analysis and the mixed effects analysis at group level is that no statistical comparison is made between the two stimulus modalities in the time series analysis. This distinction means that a higher BOLD amplitude following one stimulus modality compared to another does not necessarily mean higher activation. A statistical comparison between modalities, such as the mixed effects analysis above, is necessary to conclude that one stimulus gives more reliable activation than another.

In summary, the identification of Aδ-predominant structures based on the functional imaging data was accomplished by combining three criteria: i) mixed effects analysis, identifying main effects (at 41°C and 51°C), ii) differences in the amplitude of the responses to different stimuli (51 > 41°C and 51 < 41°C); and iii) by latency analysis of the averaged BOLD responses (i.e. single trial averages) at 41°C and 51°C.

## Results

### Evoked potentials

**Figure 2:**
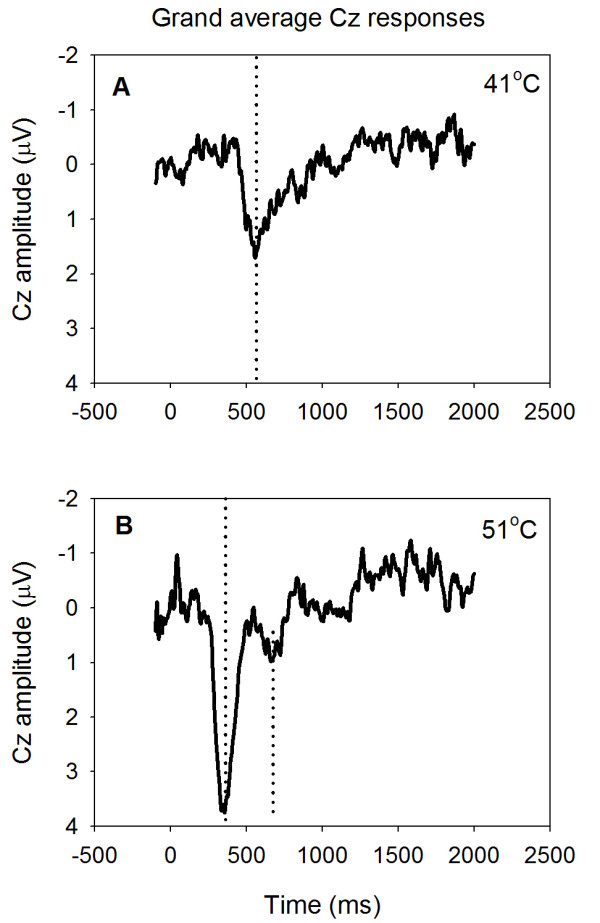
**Vertex (Cz) potentials evoked by contact-heat pulses applied to the hairy skin of the left hand**. Grand average responses were averaged across subjects after 10 stimuli delivered at 41°C (A) and at 51°C (B). Dotted lines mark the latencies of the major positive peaks (A and B).

**Figure 3:**
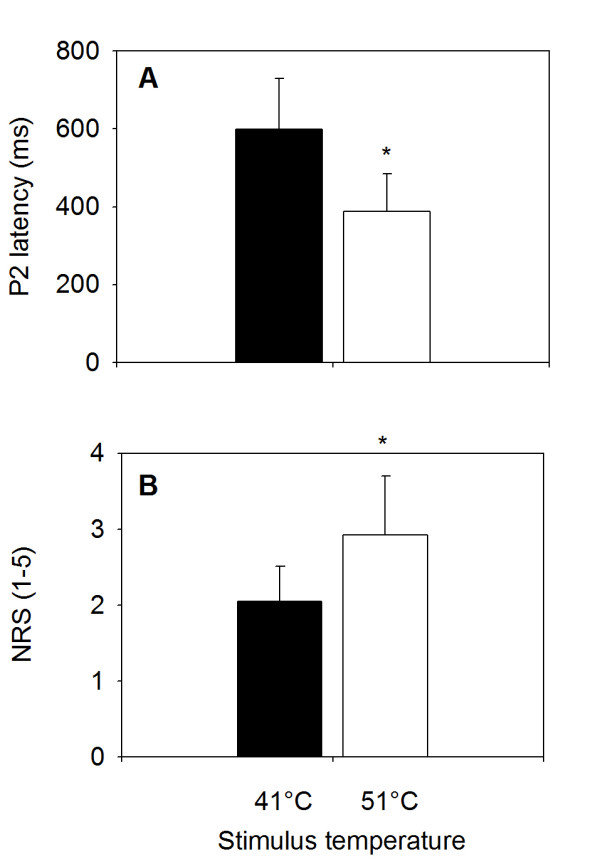
**Latency of major positive peak and pain intensity (NRS)**. A) Latency of the first major positivity in the heat-evoked potential at the vertex (electrode Cz). Latency of 51°C stimuli were significantly shorter than latency of 41°C stimuli (*p = 0.04). B) Average pain intensity ratings obtained during scanning. Ratings of 51°C stimuli were significantly more intense than ratings of 41°C stimuli (*p = 0.018). Values are mean ± standard deviation.

Contact heat evoked potentials (CHEPs) were identifiable in all but one subject. The grand average responses at Cz after ten 41°C stimuli (major positive peak latency: 554 ms) and ten 51°C stimuli (352 ms) across subjects are shown in Figure 2A and 2B, respectively. When average responses following a 41°C stimulus are quantified for each subject before averaging, the mean latency of the major positive potential is somewhat higher (598.0 ± 130.7 ms; Figure 3A). Similarly, the 51°C stimulus yields a mean latency (individual peaks averaged across subjects) of 387.7 ± 96.2 ms (Figure 3A). The difference between the two main positive peaks for 41°C and 51°C was statistically significant (p = 0.04). These latencies were similar to data presented in Granovsky et al.[14] (599.1 ± 134 ms and 405 ± 48 ms, respectively) and the latency difference is consistent with strongly predominant C fiber excitation by the 41°C stimulus and Aδ fiber excitation by the 51°C stimulus. The small, ultralate positive potential shown around 660 ms following the 51°C stimulus (Figure 2B) suggests the possibility of C fiber excitation, but this was not apparent in the psychophysical results (see below).

### Psychophysics

The average rating of the 51°C stimuli was 2.9 ± 0.8 (mean ± standard deviation; S.D.) on the 5-point NRS (Figure 3B). This was significantly higher than the average rating of the 41°C stimuli, 2.1 ± 0.5, (p = 0.018; Figure 3B). Therefore, the psychophysical results are consistent with a clear differentiation between the sharp pain associated with brief Aδ fiber stimulation and a predominantly warm, painless sensation associated with the brief excitation of C fiber activity. Subjects did not report a second-pain sensation after the 51°C stimuli.

### fMRI analysis

We sought to determine whether the cutaneous stimulation of Aδ fibers differentially activates structures that are anatomically distinct from structures activated by the cutaneous heat stimulation of C fibers.

### Group results for main effects

Of the 36 structures analyzed (small volume corrected; p = 0.01 threshold; Table 1), eight structures responded to the 51°C stimulation (three bilaterally, three contralaterally only, and two ipsilaterally only), and two structures responded exclusively to the 51°C stimulation (one ipsilateral and one contralateral), Table 2A. Eleven structures responded to the 41°C stimulation (six bilaterally, four contralaterally only, one ipsilaterally only), and seven structures responded exclusively to the 41°C stimulation (one bilateral, six contralateral, and four ipsilateral), Table 2B. Seven structures responded to both stimulus intensities (two bilaterally, three contralaterally only, and two ipsilaterally only). Thus, eight structures in Table 2A are initial candidates for being predominantly Aδ-responsive structures because they responded to the 51°C stimulus: bilateral A Ins, pACC and POFC, contralateral aMCC, hippocampus and S2, and ipsilateral P Ins and hypothalamus. The response to 51°C (Table 2A) is a necessary, but not sufficient, criterion for a structure to be identified as being predominantly Aδ-responsive, because activations in Table 2 are not based on a statistical comparison between stimulus intensities. To determine whether a structure responds predominantly to Aδ fiber stimulation, it is necessary to contrast the responses to these stimulus modalities.

**Table 2:** Main effects, by VOI, detected by mixed effects analysis

		A. 51°C stimulation					B. 41°C stimulation				
	
Structure	Side	# voxels	z-score	Coordinates (Tal)			# voxels	z-score	Coordinates (Tal)		
				*x*	*y*	*z*			*x*	*y*	*z*
Lateral thalamus	C						65	3.6	22	-12	-1
Anterior insula	C	136	3.08	30	10	0	386	3.7	32	18	5
	I	117	3.66	-32	11	-6	141	3.8	-34	16	5
Posterior insula	C						269	4.2	30	-19	5
	I	83	3.48	-42	-5	19					
POFC	C	119	3.6	40	25	2	441	3.6	42	29	-3
	C	38	3.2	24	25	-13					
	I	46	3.32	-28	21	-8	154	3.5	-28	32	-13
aMCC	C	81	2.85	10	31	35	172	3.6	6	27	34
	C						101	3.6	6	9	22
	I						113	3.7	-10	13	21
pMCC	C						18	3	10	-8	30
pACC	C	23	3.15	10	39	0					
	I	35	3.06	-10	38	18	48	3.1	-10	43	2
S1	C						86	3.5	51	-26	47
	C						34	2.8	32	-40	54
	I						76	3.5	-14	-25	49
S2	C	32	3.7	57	-17	19	103	4.8	59	-15	17
Hypothalamus	C						17	3.1	6	-12	-8
	C						19	3.1	14	0	-8
	C						32	3.1	10	1	-10
	I	17	2.67	-10	-8	-6	35	3.2	-10	-12	-8
Hippocampus	C	41	3.48	22	-28	-10	46	3.3	24	-45	-4
	C						120	3.7	22	-26	-7
	C						68	3.4	36	-14	-13
	C						70	3	22	-13	-18
	I						33	2.8	-22	-14	-18

(A) Activations during 51°C stimulation. (B) Activations during 41°C stimulation. Each line represents a cluster within the corresponding VOI. I: ipsilateral to stimulation, C: contralateral to stimulation. Coordinates are based on the stereotactic atlas by Talairach & Tournoux [19].

### Group results for contrast of stimulus responses

Table 3 shows the voxel counts and z-scores obtained from contrasting the parameter estimates from the responses to 51°C and 41°C stimuli. This contrast shows activation in the bilateral anterior insulae, the contralateral hippocampus, and the ipsilateral lateral thalamus, posterior orbitofrontal cortex, posterior insula, and perigenual anterior cingulate cortex. Two single voxel activations were in the posterior corpus callosum and not considered further. A cluster of five voxels in the ipsilateral ventricle is also not considered further. Conversely, if 51°C activation maps are subtracted from 41°C activation maps, the resulting contrast reveals structures that respond predominantly to 41°C; this includes all structures in Table 3 plus the bilateral secondary (S2) somatosensory cortex, contralateral lateral thalamus, and hypothalamus, and the ipsilateral anterior and posterior mid-cingulate cortex, dorsal posterior cingulate cortex, primary (S1) somatosensory cortex, hypothalamus, and hippocampus. A detailed presentation of these activations is not given since structures that respond predominantly to 41°C are beyond the scope of this paper. A small cluster at the border of the ipsilateral posterior insula and the opercular-temporal lobe junction appears also in this contrast but not in the main effects analysis. The ipsilateral medial thalamus, anterior insula, and caudal anterior cingulate cortex, although activated in the previous comparison, are not activated in this one.

**Table 3:** Subtraction of 41°C activation maps from 51°C activation maps

Structure	Side	# voxels	z-score	Coordinates (Tal)		
				*x*	*y*	*z*
Lateral thalamus	I	1	2.4	-10	-19	18
	I	2	2.9	-16	-27	7
Anterior insula	C	5	3.0	40	14	5
	I	1	2.5	-38	7	-7
Posterior insula	I	6	3.6	-40	-7	13
POFC	I	2	2.7	-20	23	-1
	I	3	2.6	-30	37	-5
pACC	I	31	3.7	-8	47	14
Hippocampus	C	2	2.8	26	-28	-9

Structures where 51°C activity > 41°C activity, generated by subtraction of 41°C activation maps from 51°C activation maps. Each line represents a cluster within the corresponding VOI. I: ipsilateral to stimulation, C: contralateral to stimulation. Coordinates are based on the stereotactic atlas by Talairach & Tournoux [19]. See Table 1 for structure abbreviations.

### Time series analysis

A complimentary analysis in searching for Aδ-predominant structures was to perform a time series analysis of the BOLD responses. The latency analysis identified 11 contralateral and 9 ipsilateral structures that satisfied the latency criterion (Table 4). The average peak latency was 8.0 ± 0.8 s after the 41°C stimulation and 6.7 ± 1.1 s after the 51°C stimulation (t = 4.9; p < 0.001).

**Table 4:** Peak latencies of BOLD responses by VOI

	**Contralateral**			**Ipsilateral**		
		
	**41°C**	**51°C**	**51 < 41**	**41°C**	**51°C**	**51 < 41**
**VOI**	**(sec)**	**(sec)**		**(sec)**	**(sec)**	
	
M Thal	7.5	6	*	7.5	7.5	
L Thal	9	6	*	9	7.5	*
A Ins	7.5	6	*	7.5	6	*
P Ins	7.5	6	*	7.5	6	*
S2	7.5	6	*	7.5	7.5	
pACC	9	7.5	*		6	
aMCC	7.5	7.5		7.5	7.5	
pMCC	7.5	7.5		7.5	7.5	
dPCC	9	7.5	*	7.5	6	*
M S1	7.5	7.5		9	6	*
L S1	7.5	7.5		7.5	6	*
Hy sup	7.5	7.5		7.5	7.5	
Hy inf	7.5	7.5		9	6	*
Hi sup	10.5	6	*	7.5	7.5	
Hi low sup	9	4.5	*	7.5	7.5	
Hi high inf	7.5	6	*	9	4.5	*
Hi inf	7.5	6	*	9	4.5	*
POFC	7.5	7.5		7.5	9	

Values are mean across subjects. VOI marked with * fulfills latency criterion (51°C evoked BOLD response peak earlier than 41°C evoked BOLD response).

### Aδ-predominant structures

**Figure 4:**
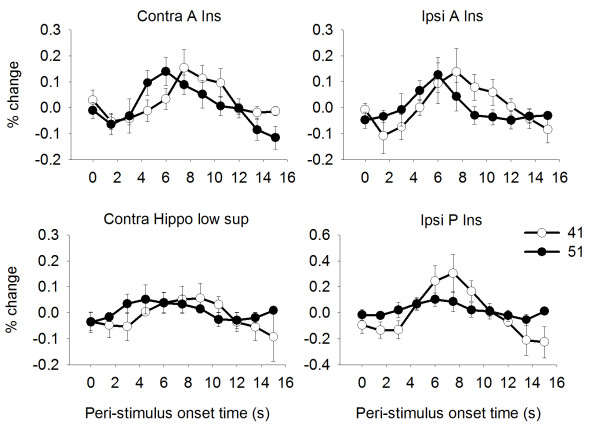
**BOLD responses of Aδ predominant structures**. Responses are from bilateral A Ins, contralateral hippocampus and ipsilateral P Ins. Each trace is the average BOLD response across subjects for 41°C (open circles) and 51°C (filled circles) ± standard error of the mean. The y-axis indicates % change from baseline (calculated as the average BOLD response over the preceding 10 s). Time resolution is 1.5 s (= TR). The 51°C BOLD peaks 1.5 - 3.0 s earlier than the 41°C BOLD in all these VOI, which is one of the three criteria established for Aδ predominant structures, as described in the text.

**Figure 5:**
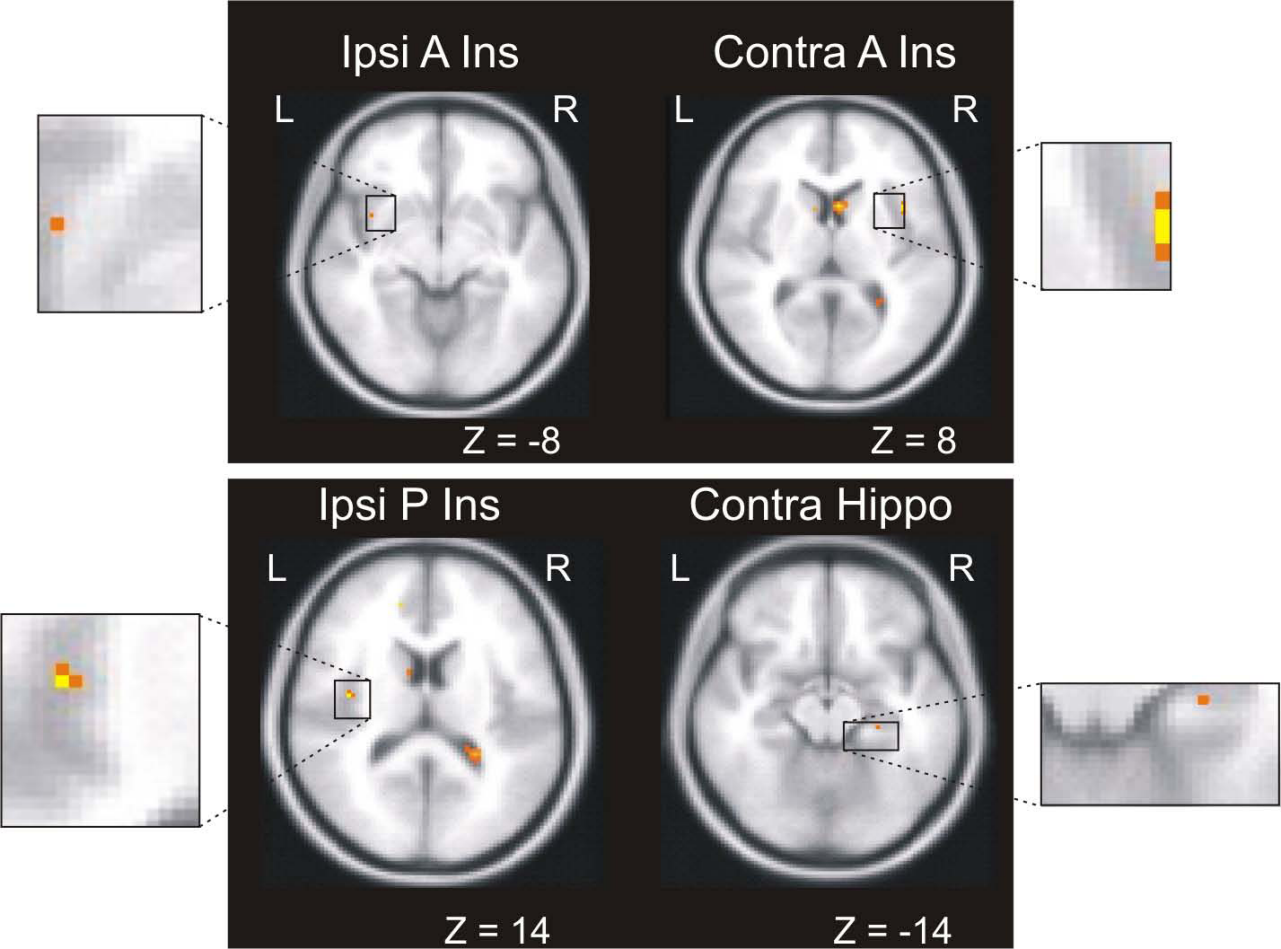
**Brain locations of Aδ predominant activation**. Activations in the bilateral A Ins, ipsilateral P Ins and contralateral hippocampus satisfied three criteria for being Aδ predominant: activation at 51°C, larger activation at 51°C than 41°C, and shorter 51°C BOLD latency than 41°C BOLD latency. Z-coordinates are in Talairach measurements.

To identify structures that have a predominant response to Aδ fiber and C fiber stimulation, we applied three criteria to our BOLD data: activation at 51°C (main effects), larger activation at 51°C than 41°C (contrast of stimulus responses), and shorter 51°C latency than 41°C latency (time series analysis). Table 5 lists the structures fulfilling each of the three criteria and the structures fulfilling all three criteria: the bilateral A Ins, ipsilateral P Ins and contralateral hippocampus. Notably, the ipsilateral P Insula fulfilled these criteria and also responded exclusively during the 51°C stimulation. Figure 4 shows the average BOLD-response to 41°C and 51°C stimulation in the structures meeting all three criteria. Please note that the time series analysis is normalized and does not allow one to conclude that a higher BOLD amplitude equals stronger activation. No statistical comparison is done between the 41°C and 51°C BOLD responses (see also Methods). The time series analysis defines a structure as Aδ predominant or not solely based on the peak latencies of the two BOLD responses (indicating an earlier processing of 51°C stimuli than 41°C stimuli). Figure 5 shows brain locations of the Aδ predominant activations. In addition to the four structures satisfying all criteria, pACC responded exclusively to 51°C by the first criterion (Table 2A).

**Table 5:** Structures fulfilling criteria 1, 2 and 3 are defined as Aδ-predominant structures

		**Criteria**			
		**1**	**2**	**3**	**1 & 2 & 3**
**Structure**	**Side**	**Grp level**	**51 > 41**	**Latency**	
	
L Thal	C			*	
	I		*	*	
M Thal	C		*	*	
	I		*		
Anterior insula	C	*	*	*	*
	I	*	*	*	*
Posterior insula	I	*	*	*	*
POFC	C	*			
	I	*	*		
aMCC	C	*			
	I		*	*	
pACC	C	*		*	
	I	*	*		
S2	C	*		*	
Hypothalamus	I	*		*	
Hippocampus	C	*	*	*	*

Structures fulfilling three criteria for being Aδ-predominant are marked with *. Criteria are 1. Significant activation at group level after 51°C stimulation. 2. 51°C activation significantly larger than 41°C activation. 3. 51°C peak BOLD latency < 41°C peak BOLD latency. C: contralateral to stimulus, I: ipsilateral to stimulus.

## Discussion

Our findings show that two distinct sets of forebrain structures participate in elaborating the perception of "first pain" that follows the preferential excitation of Aδ heat nociceptors: 1) those preferentially responsive to noxious stimulation of Aδ fibers and 2) those with similar responses to converging inputs from the innocuous stimulation of C fibers. We used cerebral potentials to identify stimuli that predominantly stimulate Aδ or C fibers. We then used these stimuli in an fMRI contrast paradigm to identify structures that were preferentially active during the pain associated with the brief excitation of Aδ heat nociceptors as compared with the innocuous warmth associated with the brief excitation of heat-sensitive C fibers. Because this image contrast reflects both heat pain and preferential Aδ excitation, the relative contribution to the contrast of each of these variables is unknown. An experiment comparing painless and painful selective excitation of both Aδ and C heat receptors could address this question but would be challenged seriously by the strong association of Aδ heat receptors with pain and the temporal summation required to evoke heat pain mediated only by C fibers [12,14,15,27]. Meanwhile, the evidence presented here strongly favors the interpretation that the bilateral anterior insula (A Ins), contralateral hippocampus (Hipp), and ipsilateral posterior insula (P Ins) play a unique and determining role in mediating the acute heat pain associated with the excitation of Aδ heat nociceptors.

### Fiber specificity of the stimuli

By selecting stimulus temperatures and measuring the latencies of contact heat evoked potentials, we assured that nociceptive Aδ fibers were stimulated preferentially by 51°C and that C fibers were stimulated preferentially by 41°C. This assessment is based on the psychophysical measurements and the CHEP results, which were essentially identical to data presented in Granovsky et al. [14] for hairy skin. We cannot completely rule out Aδ fiber contributions at 41°C, because some Aδ fibers respond to temperatures near 40°C in hairy skin [28]; however, we did not detect psychophysical or evoked potential evidence for Aδ fiber responses to this stimulus. The 51°C heat stimulation probably excited type 2, and possibly some type 1, Aδ heat nociceptors. Although heat-sensitive C afferents must be excited also by this stimulus, the only suggestion of a C fiber response was the attenuated positive wave shown around 660 ms in Figure 2B. This has been observed in some laser evoked potential studies [15,29], but the fiber type, if any, associated with this response is unknown and, in our study, there was no psychophysical characteristic associated with it. That is, the subjects did not report on a second, delayed, heat sensation after the 51°C stimuli. This observation is consistent with previous investigations [14,30] and the suppression of C fiber evoked responses following the excitation of Aδ fibers [17,31]. Furthermore, the 51 > 41 fMRI contrast excludes activations mediated by the most heat-sensitive C afferents, leaving only the possibility of some fMRI activations by high threshold C heat nociceptors that did not evoke a cerebral potential. The weak stimulation of heat nociceptors evokes innocuous sensations of warmth [32], so some of the innocuous heat stimuli we applied may have excited low threshold C heat nociceptors.

### Structures differentiating first from second pain

We did not elicit a clearly painful sensation with contact heat stimuli that evoked a cerebral potential mediated by C fibers without evidence for Aδ fiber excitation. The inability to evoke pain reliably, if at all, with single, brief C fiber selective stimuli is in accord with previous studies [9,14,17,27,29,31,33-37]. Indeed, when psychophysical measures have been obtained, these brief C fiber stimuli have been rated below pain threshold. It is notable that the phenomenon of "second pain" is by definition preceded by an Aδ fiber-mediated painful stimulus, a condition neurophysiologically quite different from a C fiber stimulus without a detectable preceding Aδ component. Furthermore, a robust and reliable elicitation of pain during selective stimulation of cutaneous C fibers is likely to require greater temporal and spatial central summation than can be achieved with the single brief stimuli used in evoked potential studies [38,39].

Because we were unable to directly compare and contrast structures active during "second pain" alone with those activated during "first pain", it is possible that two of the structures we have identified as responding preferentially during "first pain", the bilateral anterior insulae and contralateral hippocampus, would also be active during "second pain" because these structures also showed responses to the innocuous C fiber stimulus; this does not, of course, preclude their function as critical determinants in the mediation of "first pain". The ipsilateral P Ins, however, responded only during the 51°C stimulation, so the activity in this structure appears to be uniquely associated with the experience of "first pain".

#### Posterior Insula

It is not surprising that the posterior insula is differentially and perhaps uniquely responsive during what may be considered among the most intense, salient, alerting, and well localized pain experiences. The posterior insula is connected primarily with cortical areas related to somatosensory, auditory, and visual sensory functions [5,40]. The observations of Greenspan and colleagues [41] show that posterior insular lesions are associated with a significant increase in pain tolerance, but not threshold, as assessed by the cold pressor test. A small number of neurons responding to noxious stimuli have been recorded from the posterior insula of the unanesthetized monkey [42]. Single neurons responding to both innocuous and noxious somatic stimuli were localized in the more posterior granular area of monkeys [43]. Stimulation within the posterior insula evoked painful sensations at 17 of 93 (18%) insular stimulation sites of 14 patients [44]. The patients described the sensations as burning, stinging, or disabling, and located at well-defined somatic sites; these effects were more frequently elicited from the posterior than the anterior insula where visceral sensations were evoked. The pain-related region identified by these investigators overlaps the dorsal posterior insular region activated by heat pain in the functional imaging study of Craig and colleagues [45]. Indeed, the mid-posterior insula is among the most regularly responsive regions found among a variety of functional imaging studies [7,8,46].

#### Anterior Insula

Several studies suggest that the anterior insula is an essential component of the cortical network mediating early aspects of pain perception including the anticipation of pain; this is consistent with the differential, but not exclusive, response of these structures during "first pain". The anterior insula is predominantly connected with cortical areas related to limbic, olfactroy, gustatory, and viscero-autonomic functions; it receives input from entorhinal cortex, and sends projections to the entorhinal, periamygdaloid, and anterior cingulate cortices [40]. All responsive neurons in the primate anterior insula had large receptive fields to innocuous somatic stimuli; however, the investigators searched for responses with innocuous stimuli only [47]. In the study of Greenspan and colleagues [41], 2 patients with lesions involving the anterior insula had normal heat pain thresholds; and 3 patients with anterior insular sparing but involvement of both S2 and posterior insula, had elevated thresholds for heat or mechanically induced pain. In awake humans, stimulation of the anterior insula produced visceral sensory experiences and visceral motor responses, but not reports of pain [48]. However, Afif and colleagues have recently reported that cephalic pain and painful pin-prick body sensations are evoked during electrical stimulation of the middle short gyrus in the anterior, but not posterior, insula [49]. Early imaging studies revealed pain-related activity in the anterior insula [50-53], but activation during the first 10 s of repetitive noxious heat stimulation shifts from the anterior to the posterior insula as stimulation continues for 45 s [54]. Ploghaus and colleagues showed that the anterior insula was active specifically during the anticipation of experimentally induced pain rather than during the experience of pain itself [55]. Porro and colleagues [56] confirmed the relationship of anterior insular activity and pain anticipation but also found that it correlated with perceived pain intensity. The anterior insula is also among the brain structures responding specifically to stimulus novelty and salience, consistent with its differential activation during "first pain" [57]. Together with the results of our study, the observations cited above suggest that anterior insular activation is related primarily to imparting salience, arousal, and motivation to pain rather than the performance of sensory discriminative functions.

#### Hippocampus

The entorhinal cortex, the main input pathway to the hippocampus, has strong reciprocal connections with the dysgranular and agranular cortex of the anterior insula [40], providing an anatomical substrate for the conjoint responses of the hippocampus and anterior insulae during Aδ-mediated "first pain". Hippocampal activity has long been associated with mnemonic and emotional functions [58,59]. The hippocampus and entorhinal cortex may be regarded as components of a fronto-temporal cortical network for the encoding, storage, and retrieval of affectively significant sensory information emanating in part from parietal somatosensory association areas [60,61]. Although nociceptive responses of single hippocampal neurons have been reported in the anesthetized rodent [62,63], comparable studies have not been performed in monkeys or humans. Hippocampal activation has been observed in functional imaging studies of pain, especially in designs that detect responses related to unpredictability, anxiety, or fear [58,64,65]. Given the abrupt onset, intensity, and arousing capacity of Aδ-mediated "first pain", the differential response of the hippocampus is not surprising.

### Comparison with other functional imaging studies

Qiu and colleagues [12] used very brief (1 ms) infrared laser stimulation presumed to differentially stimulate Aδ and C fibers and report a pattern of overlapping activations that differs from ours (bilateral thalamus, bilateral S2 cortex, bilateral ACC, and ipsilateral mid-insula). Most notably, Qiu and colleagues did not detect any structure in which the selective stimulation of Aδ afferents evoked a unique or greater response than the selective stimulation of C fibers. There are several differences between our experiment and those of Qiu and colleagues that could explain the discrepancy: 1) we contrasted the responses evoked by painful 51°C and painless 41°C stimulation to identify activations related specifically to Aδ-mediated heat pain; 2) we used contact heat (51°C) stimulation of hairy skin, rather than an infrared laser, to stimulate Aδ fibers preferentially and; 3) verified the preferential characteristic of our stimulation by evoked cerebral potential recording and in-scanner psychophysical intensity ratings. In a related experiment, Veldhuijzen and colleagues used a diode laser to investigate the different forebrain responses to pricking and burning pain evoked by short (60 ms) and long (2 s) focused (1 mm diameter) stimuli presumably selective for Aδ and C fibers, respectively [13]. In that study, the different pain sensations were of equal intensity and unpleasantness and there was considerable activation overlap; nonetheless, a group contrast analysis revealed stronger activations during pricking pain bilaterally in the parahippocampal and fusiform gyri, in the ipsilateral hippocampus, and contralaterally in the cerebellum and occipito-parietal cuneus region. Only the ipsilateral dorsolateral prefrontal cortex showed stronger activation during burning pain. These contrasts reflect differences in forebrain processes mediating two equally intense and unpleasant pain conditions and not the activation due to pain; some of these activation differences are likely related to the excitation of different nociceptive afferents. Our experiment, however, reveals the forebrain response associated with the pain of preferential Aδ heat nociceptor excitation as compared to the painless warmth during the preferential excitation of heat-sensitive C fibers.

## Conclusions

These findings show that two sets of forebrain structures mediate the initial sharp pain evoked by brief cutaneous heat stimulation: those responding preferentially to the brief stimulation of Aδ heat nociceptors and those with similar responses to converging inputs from the painless stimulation of C fibers. Our results suggest a unique and specific physiological basis, at the forebrain level, for the "first pain" sensation that has long been attributed to Aδ fiber stimulation and support the concept that both specific and convergent mechanisms act concurrently to mediate pain.

## List of abbreviations used

aMCC: anterior midcingulate cortex; A Ins: anterior insula; BOLD: blood level oxygen dependent (response); CHEP: contact heat evoked potential; dPCC: dorsal posterior cingulate cortex; EEG: electroencephalography; EOG: electrooculography; EP: evoked potentials; fMRI: functional magnetic resonance imaging; HippHiInf: hippocampus - high inferior; HippInf: hippocampus - inferior; HippLowSup: hippocampus - low superior; HippSup: hippocampus - superior; HyInf: hypothalamus - inferior; HySup: hypothalamus - superior; LS1: lateral primary somatosensory cortex; LThal: lateral thalamus; MS1: medial primary somatosensory cortex; MThal: medial thalamus; pACC: pregenual anterior cingulate cortex; P Ins: posterior insula; pMCC: posterior midcingulate cortex; POFC: posterior orbitofrontal cortex; S2: secondary somatosensory cortex; VOI: Volumes of interest.

## Competing interests

The authors declare that they have no competing interests.

## Authors' contributions

DAM carried out the data acquisition (evoked potentials and fMRI) and drafted the manuscript. LHG contributed significantly to software programming, study design and statistical analysis of the fMRI data. TDT participated in the design and analysis of the evoked potential data. KLC participated in the design of the study, in data analysis and contributed significantly to the manuscript. All authors read and approved the final manuscript.
